# A Practical Formulary of the Parisian Hospitals

**Published:** 1830-04-01

**Authors:** 


### XLVII.

A Practical Formulary of the Parisian Hospitals, &c. &c. By R. D. M'Lellan, M.D. Duodecimo, pp. 280. Edinburgh and London, 1830.

About a fourth part of this little work is occupied with an account of the various hospitals of Paris—their medical officers—and the prominent features of their practice. The far greater part of the volume is dedicated to a copious account of the various formulæ of medicines, &c. used in the Parisian hospitals, with a copious index, facilitating reference to the whole. The work is translated from the third edition of M. Ratier.

The little volume before us will prove useful to many besides those who visit the Gallic Metropolis, though to the latter it will, of course, prove a peculiarly valuable vade mecum. An analysis is out of the question; but the notice of a single hospital will shew the manner of the translation. We shall select the

HOSPITAL FOR CHILDREN.

This institution is appropriated to the reception of children of both sexes under the age of 16 years, whatever may be the nature of their complaints. Messrs. Jadelot and Guersent are the medical officers, and, in their lectures, make known to their students, the results of their extensive experience. Patients affected with porrigo are placed under the care of two brothers, of the name of Mahon, who are proprietors of a secret remedy, which they have authority to use under the inspection of the physicians, who merely certify the cure. This, in our view of the case, is quackery, on the part of the Messrs. Mahon, although our ingenious and learned friend, Dr. Bailey of Reading, maintains that it is not so. The question might be easily settled thus:—Would Dr. Bailey keep secret a remedy which he knew to be valuable to his fellow creatures, and engross the profits of it to himself? we are quite certain he would not. The following details drawn up by Dr. Delvincourt, and corrected by M. Jadelot himself, furnish a summary of this latter Physician's opinions on a branch of medicine which he appears to practise with modesty, ability, and success.

"Long before the revolution which occurred in medicine relative to essential fevers, M. Jadelot, following the course of his observations, directed specially to the diseases of childhood, had been led to recognise in their affections a more fixed and limited seat. He did not long hesitate in referring a great number of fevers to inflammations, solitary or co-existing, of the abdomen, chest, or head. Those of the abdomen, he next ascertained, were in a proportion infinitely more considerable than those of the other cavities. On the subject of intermittent fevers, the number of which, it may be stated, is now much diminished, M. Jadelot has not formed any particular opinion. He employs the known treatment, modifying it according as he observes, amid the general disorder, any marked alteration of function in the different organs. He thus successfully adopts a mixed treatment, directing for example, antiphlogistic remedies to parts that appear to be the seat of some irritation, and at the same time, exhibiting quinquina alone, or combined with camphor, in the form of enema.

"Almost all that the class of fevers has lost, has been carried to that of the phlegmasiæ; they have been studied with more care, and their treatment, better adapted to their seat and nature, has become less tedious and more fortunate. M. Jadelot was among the first to acquire an accurate knowledge of gastric and intestinal inflammations; he created for himself, so to speak, new methods of recognising them, and arrived, by the inspection of the features of the face alone, at an accuracy of diagnosis truly remarkable. This *semeiology*, peculiar to early life, though we can sometimes derive advantages from it in more advanced years, M. Jadelot has named physiognomonic: in a very few words, a succinct idea of it may be afforded to the reader. Independently of the alterations of colour which the face presents, and which furnish to the physician signs more or less positive, the expression of the physiognomy, and the prominence, more or less considerable, of particular traits, are also means of clearing still farther the diagnosis.

"In the infant, whose facial muscles are not endowed with great mobility, three principal traits present themselves to the observer. They are nearly parallel, and direct themselves from the centre towards the sides of the face; the first, *(oculo-zygomatic,)* proceeding from the great angle of the eye, loses itself a little below the projection formed by the cheek-bone; the second, *(nasal,)* commences at the superior part of the wing of the nose, and prolongs itself, in a semicircle, towards the commissure of the lips; it is sometimes crossed by a small trait (*genal*) which directs itself towards the middle of the cheek; the last, (*labial,*) arises at the commissure of the lips, and loses itself towards the chin.

"Each of these traits is in relation to one of the visceral cavities. The first is connected with the diseases of the cerebro-nervous system; the second, and its accessory, (the *genal,*) with abdominal lesions; the third, lastly, indicates the affections of the organs of circulation and respiration.

"This mode of investigation, valuable in regard to patients incapable of furnishing any clear information to the physician, requires much experience; but the results obtained from it indemnify the physician for the trouble to which he submits, in order to gain his object.

"In variola, M. Jadelot endeavours particularly to ascertain the different affections, manifest or latent, with which it may be complicated, and on this knowledge he modifies his treatment. Thus, when a violent angina shews itself, without having regard to the period of the eruption, he employs antiphlogistics, and the most powerful derivatives. If symptoms of gastric irritation supervene, he combats them by the appropriate means, assured that the eruption will proceed with more facility in its proper course. In the case of confluent variola, he has recourse to excitants, and even to the internal use of tonics, either administered by the mouth, or in enemata. During the period of suppuration, he recommends to let the pus contained in the pustules flow out, especially when collected in large quantity from the confluence of the pustules, by opening them with the point of a lancet, or by cutting with scissors the top of the pustules, which are afterwards cleansed with a bit of fine linen. During convalescence, he directs the simple or emollient tepid bath, to hasten the fall of the crusts, and facilitate the cutaneous transpiration.

"Rubeola is still more frequently complicated than variola, with inflammation, either of the mucous or parenchymatous structures of the thoracic organs, and this complication merits as much, and even more, care than the original disease. To antiphlogistic measures, M. Jadelot is accustomed to add, as tending to diminish the pulmonary congestion, warm hand baths, (*maniluves,*) rendered more stimulating by vinegar, common salt, or the flour of mustard. If the eruption, after having appeared, is suddenly suppressed, which usually happens on inflammation arising in another part of the system, he has recourse to measures proportioned in their activity to the danger which threatens the patient. If the eruption, notwithstanding, be slow in re-appearing, he employs the vapour bath, or frictions, either dry, or with the addition of stimulating liniments. He does not hesitate to prescribe both tonics and stimulants, when, on account of the weakness of the subject, the eruption does not maintain its proper character and course. He confines convalescents to a pretty severe regimen, and administers purgatives but seldom, and those only of the mildest kind.

"The treatment of scarlatina differs little from that of measles. M. Jadelot recommends us to examine carefully the state of the throat, in order that we may be able to oppose in time the angina gangrenosa, which often comes to complicate the disorder.

"The angina gangrenosa is identical, whether it be preceded by scarlatina, or manifest itself without previous disease. At the commencement of this affection, which is generally inflammatory, M. Jadelot follows a treatment purely antiphlogistic; but when he perceives, in the bottom of the throat, broad whitish spots, accompanied with signs of general debility, he has immediate recourse to sinapisms to the feet, gargles, consisting of a decoction of cinchona, cataplasms of the flour of rice, prepared with the same decoction, and sprinkled with aromatic vinegar at the moment when applied to the throat, enemata of the camphorated decoction of bark, diluent drinks, and fumigations with vinegar, directed towards the bottom of the throat. He favours the action of the bark cataplasms by frictions, made on the sides of the neck, with liniment of ammonia. Sometimes, though rarely, it is necessary to resort to the internal administration of tonics.

"Inflammation of the mucous membrane of the air passages is a very frequent disease of infancy, and the nearer it is to the larynx, its severity is the more obvious. M. Jadelot is the first who attained, by an attentive examination of the patient, a knowledge of the precise seat of the angina, a circumstance of great importance as to the treatment, which ought to be conducted with more or less activity, according as the larynx or trachea is affected, or rather according to the extent of the inflammation in the lining membrane of the air tube. This treatment consists in the application of leeches to the fore-part of the neck, in sinapisms to the inferior extremities, laxative or purgative enemata, and emetics. He gives the preference, in general, to ipecacuanha in powder or in syrup, and employs it in the case of weak subjects, of a sluggish system, after having abated the inflammation by topical bleeding. He entertains, in such cases, no fear from its frequent repetition, even at short intervals.

"M. Jadelot considers croup as a species of angina of the air passage, offering more violent symptoms, and having, as its special character, veritable paroxysms, divided by well marked remissions. He admits of different degrees in the disorder with regard to intensity, but without changing his opinion respecting its nature.* Emetics, and the abstraction of blood by leeches, are the remedies chiefly employed in the treatment of croup. An emetic alone has often sufficed to check the disease, especially in weak pale subjects; but in other cases, he insists on the application of leeches,† and encourages the flow of blood, until the child become pale, or the force of the pulse much diminished. If the bleeding be suspended too soon, we run the risk of not arresting the evil, and are obliged, as the troublesome consequence of this, to re-apply the leeches. After the bleeding, an emetic is given, and vomiting kept up at intervals of two or three hours, and this practice is attended with the greatest success, the children finding relief after each act of vomition.

"When croup has reached the second stage without having been subdued, and the presence of a false membrane is suspected, M. Jadelot again applies leeches. As soon as they have fallen off, he excites vomiting, and in this case he employs, in spoonfuls every ten minutes or quarter of an hour, the potion termed *anti-croupale*, until the effect of vomiting is obtained. He equally insists on the use of derivatives, which act on the skin or intestinal canal; and recommends also to provoke sneezing as a means of detaching the false membrane from the trachea.

"As to the question whether, when the disease is very rapid, we should commence by blood-letting or by an emetic, M. Jadelot thinks that we should first bleed if the child be robust, and present symptoms of congestion towards the superior parts; but, on the contrary, that an emetic should precede blood-letting, which can after-

---

* "M. Guersent, in his clinical lectures, notices a *false croup*, which is cured without any other means than diet, rest in bed, and demulcent drinks.

† "M. Jadelot has never been deterred by the fear of being unable to stay the flow of blood from the punctures made by the leeches, in a part where the vessels are numerous, and in which compression cannot be exerted. This can always be accomplished with agaric immersed in vinegar, or powdered with alum or colophony, or, finally, by means of the bluestone. In order to apply it suitably, it is necessary to dry the bites, and as soon as there is formed a small blackish crust, to cover it with a small piece of agaric supported by the pressure of the finger for a few minutes. Lastly, cauterization, by means of a small stylet, raised to a white heat, of all the methods is the most sure, and least painful.

wards be practised, when the subject is pale, and when little heat or fever exist.

"The sulphuret of potass is a medicine which he rarely employs in croup, and when the inflammation runs high he regards it as dangerous. The dose varies, but it ought not to exceed half a drachm in the twenty-four hours.

"After numerous observations, M. Jadelot believes he has ground for the opinion, that pertussis consists in an inflammation of the bronchia, associated with a particular lesion of the nerves, which distinguishes it from ordinary catarrhs. In consequence of viewing the disease in this light, the treatment consists in blood-letting, relaxants, exhibited in various forms, employing at the same time derivatives* and narcotics. Besides the internal use of narcotics, their external application to the thorax is also directed, which is done by sprinkling a cataplasm with a drachm or two of pure laudanum, or with a solution of the extract of opium.

"In hydrocephalus acutus, M. Jadelot distinguishes a gastro intestinal irritation which shews itself at the outset of the disease, and which precedes, more or less, the development of the cerebral symptoms, and to which he opposes topical bloodletting, with emollient applications to the abdomen. When the head, subsequently, appears to be the principal seat of the affection, he adopts antiphlogistic measures, with an immediate view to the condition of this organ; without losing sight, however, of the abdominal irritation. He does not apply ice to the head, except in the first stage of the disease, before effusion has formed, and when there exists a violent congestion towards the brain. He advises us not to have recourse to this application till after the necessary local bleeding has been premised, and to employ it while the patient is placed in a tepid bath. Such are the means used in the first stage; but when the symptoms have made known the existence of effusion beneath the cranium, M. Jadelot resorts to external derivatives: he applies a blister to the nape of the neck, and orders frictions of the extremities, with acetic æther, or with volatile and aromatic liniments, mercurial frictions to the shaven head, repeated every three, four, or six hours, after having washed the head in the intervals with some ammoniacal liniment. He administers, at the same time, calomel internally, in the dose of two, three, or four grains, repeated four or five times a-day. Finally, the extreme resource is a very large vesicatory to the head."

* "Sinapisms of such strength as only to produce a slight redness, and often renewed. Frictions on the arms and chest, with acetic æther. Towards the end of the disease, good effects are obtained from blisters."

Whether M. Ratier or the translator be in fault, we have not time at present to inquire; but we suspect that there are occasional mistakes in the quantities of the medicines prescribed in the formulæ which ought to be carefully re-examined. The two following prescriptions must surely be erroneous.

TONIC MIXTURE OF DUPUYTREN.

℞. Extracti Cinchonæ .... ʒij.
Extracti Opii .......... ℥ss !!
Syrupi Cinchonæ Vinosi, ℥ijss.
Aquæ Menthæ ........
Aquæ Canellæ ......āā ℥iv.
Misce.

Thus, in a mixture of eleven ounces, there would be 240 grains of extract of opium!!

The next prescription which we glanced at was the ointment of iodine, in which half an ounce of the hydriodate of potash is ordered to be combined with an ounce and a half of lard. This is infinitely too much, and would immediately irritate the skin when applied in friction.

We do not suppose that these errors are numerous, though they appear somewhat startling. The formulæ should be carefully revised, and a table of errata prefixed to the volume, when it will no doubt prove a very useful publication. We shall notice some detached subjects in other departments of our Periscope.

